# Can an electronic monitoring system capture implementation of health promotion programs? A focussed ethnographic exploration of the story behind program monitoring data

**DOI:** 10.1186/s12889-020-08644-2

**Published:** 2020-06-12

**Authors:** Kathleen Conte, Leah Marks, Victoria Loblay, Sisse Grøn, Amanda Green, Christine Innes-Hughes, Andrew Milat, Lina Persson, Mandy Williams, Sarah Thackway, Jo Mitchell, Penelope Hawe

**Affiliations:** 1 The Australian Prevention Partnership Centre, Ultimo, Australia; 2 Menzies Centre for Health Policy, School of Public Health, Faculty of Medicine and Health, University of Sydney, Sydney, Australia; 3 University Centre for Rural Health, Faculty of Medicine and Health, University of Sydney, Camperdown, New South Wales 2006 Australia; 4 Technical University of Denmark, Engineering Systems Group, Innovation Division, Department of Technology, Management and Economics, Kongens Lyngby, Denmark; 5 Office of Preventive Health, Sydney, Australia; 6 Centre for Epidemiology and Evidence, New South Wales Ministry of Health, Sydney, Australia; 7 Health Promotion Service, South Western Sydney Local Health District, Liverpool, Australia; 8 Centre for Population Health, New South Wales Ministry of Health, Sydney, Australia

**Keywords:** Health promotion, Performance monitoring, Prevention, Obesity, Implementation science, Ethnography, Health policy, Health management, Scale up

## Abstract

**Background:**

There is a pressing need for policy makers to demonstrate progress made on investments in prevention, but few examples of monitoring systems capable of tracking population-level prevention policies and programs and their implementation. In New South Wales, Australia, the scale up of childhood obesity prevention programs to over 6000 childcare centres and primary schools is monitored via an electronic monitoring system, “PHIMS”.

**Methods:**

Via a focussed ethnography with all 14 health promotion implementation teams in the state, we set out to explore what aspects of program implementation are captured via PHIMS, what aspects are not, and the implications for future IT implementation monitoring systems as a result.

**Results:**

Practitioners perform a range of activities in the context of delivering obesity prevention programs, but only specific activities are captured via PHIMS. PHIMS thereby defines and standardises certain activities, while non-captured activities can be considered as “extra” work by practitioners. The achievement of implementation targets is influenced by multi-level contextual factors, with only some of the factors accounted for in PHIMS. This evidences incongruencies between work done, recorded and, therefore, recognised.

**Conclusions:**

While monitoring systems cannot and should not capture every aspect of implementation, better accounting for aspects of context and “extra” work involved in program implementation could help illuminate why implementation succeeds or fails. Failure to do so may result in policy makers drawing false conclusions about what is required to achieve implementation targets. Practitioners, as experts of context, are well placed to assist policy makers to develop accurate and meaningful implementation targets and approaches to monitoring.

*“The tick in [the PHIMS information system] is like the tip of an iceberg. It's that tiny bit above the surface. And behind it is years of chatting, visits, gently urging, suggesting they go in this direction rather than that direction.” –* Quote captured in ethnographic fieldnotes, Team G

## Background

There is broad agreement that policy-level investment in population-level health promotion is needed and effective in keeping people healthy and reducing health costs [1, 2]. But health promotion has often struggled to maintain funding and political support [3]. Policy makers and population-level program coordinators are therefore faced with a pressing need to demonstrate progress on investments in health promotion. Information technology (IT) systems hold promise to assist policy makers in tracking delivery of population-level health programs and the achievement of implementation targets, but there are very few examples of their use in population health contexts [4].

In clinical contexts, IT systems have a long history of design, use, and often, abandonment [5, 6]. The design, implementation, success and failures of electronic health records, for example, constitute a wealth of experiences, evaluation and research. This history provides a rich source of material by which to inform, debate, and interrogate the value, design, and standards for best use and implementation of electronic health records [7, 8]. In the context of using IT systems to monitor the implementation of population-level health programs these conversations are only just beginning. There are signs that current systems designed for research purposes fail to translate to everyday practice [9]. And that some systems designed for monitoring health promotion and prevention programs are overly onerous, resulting in push-back from users and, ultimately, abandonment despite considerable financial investments [10].

As more health promotion programs are scaled up to be delivered at the population-level, the demand for IT systems to monitor implementation will increase. Such systems need to effectively capture and monitor implementation progress for coordination across sites. However, processes by which to effectively monitor, implement and sustain programs at scale are understudied [11, 12]. This includes research on monitoring systems for health promotion programs delivered at scale, particularly because there are very few examples of these IT systems to study [4].

In New South Wales (NSW), Australia, the Ministry of Health designed and rolled-out an over AU$1 million IT implementation monitoring system, Population Health Information Management System (PHIMS), to facilitate and track the reach and delivery of state-wide childhood obesity prevention programs called the Healthy Children Initiative (HCI) [13]. PHIMS is unique in that it has sustained implementation since 2011, whereas many IT systems – in clinical and population health- have failed [4]. It presents a unique opportunity to examine the use of IT systems to track large-scale implementation. We set out to explore what aspects of implementation are captured via this IT monitoring system, what aspects are not, and the implications for future IT implementation monitoring systems. Our purpose was to gain insights that might improve coherency between what a recording system captures and what it really takes to achieve implementation.

The findings in this paper are part of a larger study that examined the dynamics between PHIMS use and health promotion practice [14]. The Monitoring Practice study was co-produced via a partnership with state-level policy makers and program managers for HCI, PHIMS technical designers, health promotion practitioners, and university-based researchers – all of whom are co-authors of this paper. While the university-based researchers (hereafter called ‘researchers’) led the collection and analysis of data, the roles played by the wider co-production team served to help position and interpret the findings within the broader context of HCI implementation (more information about the roles and contributions of team members are in Additional file 1). The research questions guiding this analysis were developed with the partners at study’s outset and are:

1. What constitutes/is the *breadth* of work involved in supporting early childhood services and primary schools to achieve HCI practices?
2. What constitutes/is the *intensity* of work?
3. To what extent are *breadth* and *intensity* captured by PHIMS?

### Context

PHIMS was designed to support the implementation of the Healthy Children Initiative (HCI) – Australia’s largest ever scale-up of evidence-based obesity prevention programs, which includes programs delivered in approximately 6000 early childhood services and primary schools across NSW. Two particular HCI programs, Munch and Move and Live Life Well at School (hereafter referred to as HCI), are delivered by 14 health promotion teams that are situated within 15 local health districts across the state. To date, these HCI programs are currently reporting high rates of participation, reaching over 89% of early childhood services (3348/3766) and 83% of primary schools (2133/2566) [15].

The PHIMS system is described in detail elsewhere [15, 16]. It is used to track the adoption of healthy eating and physical activity practices in schools and services (See Additional file 2 for full list of practices) for Munch and Move and Live Life Well at School. Site-level progress is aggregated via PHIMS and enables district and state-level coordinators to track district-level progress against key performance indicator (KPI) targets. Access to PHIMS is restricted via a state-wide login service, where user access is configured according to roles. User roles include 1) health promotion practitioners who access and input notes and record progress towards implementation targets for their assigned sites; 2) supervisors who can access all information entered by their staff for sites in their health district; and 3) state-level managers who can only access aggregated data reporting progress towards KPI target achievement at the health district level.

## Methods

### Data collection

Our approach to data collection was consistent with focussed ethnography. Focussed ethnography is a short-term, high-intensity form of ethnography where short visits to the field (or, relatively short in comparison with traditional ethnography) are balanced with extensive preparation, focussed selection and attention on specific activities and sites relevant to the research questions, use of multiple data sources, and intense, iterative, and collaborative analysis of data [17, 18]. Preparatory work for this study began 1-year prior to field visits during which time researchers conducted informal interviews with study partners, attended PHIMS demonstrations and reviewed documentation, met with sites to discuss the approach, and conducted a thorough review of a range of theories as a sensitisation tool [14].

The fieldwork was conducted with all 14 local health promotion teams funded to deliver HCI programs across NSW. Over 12 months, three researchers (KC, VL, SG) spent between 1 and 5 days in each health district observing the day-to-day implementation work conducted by health promotion practitioners who delivered the HCI programs. Researchers collected extensive field notes, pictures, recordings from ad hoc interviews, and program materials that were compiled in an NVIVO project database [19]. In total, we shadowed, interviewed or observed 106 practitioners across all 14 teams. Researchers recorded their observations as soon as possible after leaving the field yielding over 590 pages of detailed field notes. Regular meetings between the researchers enabled iterative, theory-informed dialogue and analysis and reflection on the interpretations arising through analysis. Ongoing correspondence with participants in the field and regular meetings with the broader co-production team allowed the representation of findings in field notes to be further critiqued and interpreted. This group approach to analysis and interpretation reduces the subjectivity of field notes by enabling interpretations to become shared by those they are about, rather than the purview of a lone ethnographer [17]. While raw field notes were only shared among the researchers and sometimes with participants whom they were about, only de-identified or abstracted data were shared with the broader co-production team and in public forums. We used the consolidated criteria for reporting qualitative research (COREQ) guidelines [20] to guide the reporting of our study (see Additional file 1).

### Data analysis

We used a grounded theory approach to code the materials in the project database, and to generate an initial project codebook, as described more fully elsewhere [14, 21, 22]. For this subsequent analysis, we used both a deductive and inductive approach [23]. Two researchers (KC and LM) collaborated in an iterative process of coding, reflection, and theming the data. For the deductive analysis, we extracted data from 15 codes we determined were most relevant to the research questions (see Additional file 3 for list and description of codes). Using a directed content analysis approach [24], we recoded this data to develop new codes related to “breadth” and “intensity,” as defined a priori (see definitions in Table 1) and generated a list of activities, strategies and resources that reflected the breadth of HCI activities. An initial coding scheme was developed and operational definitions for each category were defined and iteratively revised. We revised our coding list and recoded the data until no new codes emerged and theoretical saturation was reached. Through this process, we produced a rough ‘taxonomy’ of categories of activities involved in HCI implementation.

**Table 1:** Definitions of “breadth” and “intensity” as operationalised in this analysis

Term	Definition
Breadth	The range & type of activities, strategies and/or resources involved in day-to-day implementation work by health promotion practitioners in delivering the HCI programs
Intensity	The amount of time and effort these activities take (e.g. duration and frequency of the activity and how many steps involved in completing an activity) and the value placed on the activity by practitioners

Whilst this approach worked well for research question 1 regarding “breadth,” we found few examples of “intensity” using this approach. It was difficult to observe and interpret “intensity” in our field notes, and because our study was cross-sectional, we were unable to fully observe “intensity” in practice. However, many practitioners discussed this issue so we adopted a grounded theory approach to better explore what “intensity” of work looked like in our dataset. Through an iterative process of discussions with the ethnographers, using NVIVO tools to expand coding to the broader context, and reading many field notes in their entirety, we inductively developed codes by coding data line-by-line, and subsequently looking for overall patterns.

To answer the third research question, we coded data that spoke to two overarching, abstracted questions throughout the entire coding process: a) “Is a tick in PHIMS a true reflection of the work done?”, and b) “How is breadth/intensity of work captured in PHIMS?” The second question was not an analysis of PHIMS content, but rather, what we observed or learned about PHIMS during our field work. Therefore, to answer this question we drew both on data from ethnographic field notes, memos created during coding, and our knowledge of the PHIMS system developed over the course of the full project (e.g. through demonstrations of PHIMS, training manuals, conversations with PHIMS developers, etc). The lead authors met frequently during the coding process to discuss emerging insights, as well as met with the other researchers to discuss possible interpretations and interesting examples. Through this iterative process, we moved from concrete codes that are descriptions of activities to more abstract, thematic groupings and generalizations which we report in our results, along with a thematic conceptual model (presented later) [25].

The analyses were concurrent, occurring alongside regular meetings with the full research team and partners where emergent findings and interpretations were discussed and collaboratively explored. Coding and insights therefore developed iteratively whilst project meetings enabled feedback and reflection to be incorporated as part of the analysis process. We presented initial findings to partners for comment and reflection.

## Results

### “Breadth” of work to implement HCI

We sorted activities used by teams and practitioners to deliver HCI into 15 groupings (see Table 2). These groupings reflected two overarching purposes: 1) work involved in the implementation of HCI; and 2) work required to convert implementation work into PHIMS data. We explore these activities and how they are captured in PHIMS below. The groupings were not mutually exclusive, with specific activities often meeting multiple purposes (e.g. site visits are used for networking, distributing resources, team work, and other purposes). The types of activities within each grouping were diverse and implemented differently across teams. For example, we observed some teams devoting many hours and financial resources to activities categorised as “developing HCI resources” however, others drew mainly from centrally distributed HCI materials. Notably, there was no one way to implement HCI. Practitioners were aware that HCI implementation activities differed from team to team. They were notably curious to learn from the researchers how their approach matched or differed from other teams.

**Table 2:** The breadth (range & types) of work involved in the implementation of the Healthy Children Initiative and how it is recorded in PHIMS

The range and types of activities involved in the daily implementation of HCI	PHIMS functionality for capturing this work^a^	Variability in approaches by PHIMS users^a^
**1. Work involved in implementing HCI programs and practices**		
**Development of HCI resources and materials:** The resources produced and distributed by practitioners to support the delivery of HCI programs (e.g. factsheets, newsletters, Facebook and social media accounts, questionnaires)	No specific function to record work involved in resource development and distribution in PHIMS.	Some practitioners choose to enter notes about materials distributed^a^. No observed instances of users using PHIMS to document resource development.
**HCI Pre-work:** Work that builds the foundation for implementation of program practices (e.g. onboarding new sites, action planning, getting sign-off, data agreements and consent from sites to collect implementation data)	Site details are loaded into PHIMS by central management either through database updates, or upon request from users. PHIMS has ability to keep record of contacts details including contact information and training status of active sites and staff.^a^	Most work about practitioners’ process to recruit and onboard sites prior to becoming ‘active’ in PHIMS is recorded in alternate systems.
**Site visits:** Work involved in preparing for and conducting a site visit, including scheduled follow-ups.	Free-text boxes in PHIMS allow users to enter notes about site visits. PHIMS does not have functionality to quantify the work involved, including the time it takes to complete the site visit. PHIMS has “alert” functions for scheduled follow-ups with sites at 1, 6- and 12-month intervals. If a site visit is not documented in PHIMS within a specified time window, the practitioner and their supervisor are notified.	All teams enter data to record practice achievement; some teams have a dedicated PHIMS ‘champion’ to record this data, whereas in others, each practitioner is responsible for entering data on their sites. The amount of detail entered about the site visit varies depending on individual practitioners.
**Team work:** How HCI teams work together to conduct the work and achieve implementation targets (E.g. collaboration and team work within HCI teams, or with other districts)	Site details and notes can be shared among team members at the supervisor’s discretion.	Some practitioners keep detailed notes in PHIMS to provide their team with a full overview of the site and to keep a record for other practitioners^b^.
**Workshops (in-services):** Work involved in organising, and hosting in-services (training workshops with teachers in order to teach them and meet the training requirements for certain practices)	PHIMS allows user to schedule ‘training’, to mark invitations sent and to mark training completed. Workshop attendees are entered into PHIMS individually, and each recorded as trained. PHIMS also has a function to update training status of multiple users or sites in-bulk. There is no function to record other types of workshops, e.g. hosted to support general program delivery.	As above, and practitioners use PHIMS to record training status. Some practitioners also include qualitative notes about how the workshop went, and how much progress they made^a^.
**Organisational work:** Basic work tasks required to support and keep track of HCI work (E.g. keeping notes on scheduled follow ups, saving emails, cancellations and reorganising site visits)	Scheduled follow-ups are specifically designed to facilitate organisational work by providing a record of due dates and reminders. PHIMS has rudimentary functionality to send/save emails.	Some practitioners cut and paste emails with contacts into PHIMS’ ‘additional contact notes’ – a free form note taking data field^a^.
**Networking, communication and relationship building with sites:** The extent of networking, communication and building relationships required with sites in order to satisfy a tick (E.g. how practitioners interact with sites (emails, phone calls), build relationships with contacts and work in partnership with sites)	As above. PHIMS has ability to keep record of “contacts” (e.g. phone calls, emails) with site contacts.^b^ PHIMS has functionality to do bulk updates for multiple sites at once, which can be used to record the distribution of resources (newsletters) to all services or when an practitioner has phoned/emailed all their services to provide information or invite them to a training/information session.	Practitioners use the contact details for sites contacts. Some practitioners also record details about their interactions with sites.
**Network meetings:** Work involved in coordinating and running network meetings. Network meetings are used to engage and have more contact with site staff, offer a support network and may provide them with training that helps meet implementation targets	Practitioners can input training dates, invitations sent and whether sites attended trainings, which are often held in conjunction with meetings. It cannot track sites' registration.	Network meetings are used by some practitioners to collect information on practice adoption and update quantitative implementation data in PHIMS.
**Strategic work:** Work done to achieve implementation targets or implement practices in a strategic manner. (E.g. how the team makes decisions, weighs options, plan and uses resources to achieve targets)	PHIMS reports (including customizable reports) are available to assist with strategic work.	Use of PHIMS reports varies dependent on skill of users and teams. Some teams with superusers generate bespoke, detailed reports while others use this function sparingly, if at all.
**Flexible work to support HCI work and practice implementation:** Work involved in the development of new materials that assist in achieving HCI practices. (E.g. an app to help teach fundamental movement skills, an informational handout, school veggie gardens)	No specific function	Some practitioners may document this work in notes^a^
**Self-directed work of HCI Team members:** Work tasks/activities that practitioners choose to do or have special interest in that may or may not align with HCI program goals	No specific function	Some practitioners may document this work in notes ^a^
**Work that addresses a site’s self-identified needs:** This work may or may not align with achieving a particular practice, and may be responding to a sites’ need or request that differ from the aims of HCI	No specific function	Some practitioners may document this work in notes^a^
**2. Performance monitoring work required to convert the work done into PHIMS data**		
**Interpreting what program adoption looks like**: Collaborative or deductive work to interpret practices to know what practitioners must report on	PHIMS lists each practice that must be reported against (see Additional file 2 for the practices). It does not provide interpretative guidance, but a monitoring guide is available to assist with interpretation.	Some teams have internal discussions to interpret implementation targets and determine a consistent minimum standard for ticking a practice. We didn’t observe these conversations being documented using PHIMS.
**Collecting implementation data in sites:** How practitioners go about collecting information on practice achievement	Practice achievement status is recorded in PHIMS via a multiple-choice survey. PHIMS provides a printable template for data collection	PHIMS is not available via mobile devices and is difficult to access from non-team computers, so data entry is usually done in the team office.
**Data entry in PHIMS:** Inputting information from site visits, and other HCI activities into PHIMS to record progress and achieve a tick (i.e., target practice adopted in the site)	PHIMS has “alert” functions for scheduled follow-ups with sites at 1, 6 and 12 month intervals. So, if this data is not recorded already, the practitioner is advised.	We did not observe that PHIMS records information about user behaviour (e.g. active time spent on PHIMS, number of log-in or date of last log-in).

*PHIMS* Population Health Information Management System, *HCI* Healthy Children Initiative

^a^PHIMS contains a ‘notes’ function with a limited character allowance that users may use at their discretion. Practitioners may use this functionality to record information about the types of activities in implementing HCI. We have noted instances where we observed this function being used to record tasks or instances where we expect it might be used. However, the notes function lacks search and retrieval functions to be able to thoroughly assess content

^b^Note that the ability to *record* this information and the ability to later *retrieve* it in a useful and meaningful way is an important distinction

#### Work involved in implementing HCI

This category represents work tasks that constitute foundational components for delivering HCI to achieve implementation targets. Many of the activities were observed across all teams, but there was diversity in the specific tasks and styles by which individual activities were implemented. For example, “site visits” constituted a large proportion of practitioners’ work in every team, but approaches varied with some teams conducting regular, in-person meetings whilst others rarely visited in-person or conducted visits electronically, i.e. via phone or email (variations will be discussed in more detail later).

Some practitioners described doing work with sites who had already achieved 100% of their implementation targets. The sense from practitioners was that this responsive or self-directed work was different from or in-addition to the main work involved with implementing HCI. Sometimes this work was done to maintain practices and achievements. We also observed practitioners taking on projects or activities in response to needs identified by the local community or HCI site (e.g. to better reach culturally and linguistically diverse communities). Often – but not always- this work complemented the HCI program aims. For example, in one site practitioners developed a smartphone app to help teach fundamental movement skills. In another, they developed vegetable gardens. Not all practitioners had scope to innovate within their roles, but those that did explained that innovating was significant in keeping things fresh and new for practitioners and for sites.

##### How work is captured in PHIMS

The extent to which information about breadth was captured in PHIMS varied by category (see Table 2). For some categories, specific PHIMS functions facilitated documentation. The most widely observed and frequently used function was recording details about site-level progress in adopting practices. But beyond this, data entry in PHIMS highly varied depending on the style and skill of individual users and teams. Most practitioners, but not all, used PHIMS-specific functions to track contact details of key contacts. Also, almost all practitioners received the inbuilt reminders to schedule site visits, but their views on the value of this feature varied. PHIMS provided advanced functions – such as developing reports – but only few individuals used these.

But for many activities, such as developing educational resources, PHIMS lacked the necessary tools to enable practitioners to document their work. This was particularly true of self-directed work or work undertaken with sites that may not align directly with HCI program goals. We observed that many practitioners used the generic “notes” function to document details about how they used or distributed specific resources. The “notes” function is a free-text entry box with a limited character limit. There were multiple “notes” sections in different parts of PHIMS, however, that posed problems in summarising, searching and retrieving information. This limitation created confusion about how to best use the “notes” function to retrieve data to support implementation.

#### Performance monitoring work required to convert the work done into PHIMS data

This category represents work done to translate information about HCI implementation into data in PHIMS. Teams spent considerable time recording sites’ ongoing progress and their activities into PHIMS. Converting work into data in PHIMS required practitioners to interpret what practice adoption looks like, and to collect and translate information on sites’ practice adoption into PHIMS records.

##### How work is captured in PHIMS

PHIMS provides a user dashboard that tracks team-level progress towards team-level implementation targets. We did not observe that practitioners could extract information about how they used PHIMS. Such information might include, for example, date of last log-in, or active time spent working in PHIMS. Practitioners told us that supervisors have access to user-level data, but we only observed a few instances in which they accessed this information. We learned that supervisors can assign sites to practitioners, can access practitioners’ records, and receive information about target achievement and whether sites visit data were entered within specified time frames.

### Intensity of work to achieve an implementation target

Four interrelated themes emerged in relation to intensity. Sub-themes are underlined in the text below and correspond to illustrative extracts from the field notes presented in Table 3.

**Table 3:** The “intensity” of work involved in implementing the healthy children’s initiative, themes and exemplars from ethnographic fieldnotes

Theme	Excerpts from ethnographic field notes
***The difficulty of particular practices***	
Multi-component practices	They [this HCI Team] don’t invest money in this [practice] because it is so hard to achieve all three parts, and they don’t get credit for only achieving a portion of it. –Team I
Practice achievement is influenced by broader factors outside of an individual’s control	[The health promotion officer] gives an example of a rural area where their local business– a general store/bakery – provides the canteen 1 day a week. The school doesn’t have any other resources - parents, volunteers, facilities etc. - to provide the canteen so working with the local business is the only way to get food into the school. So they don’t have a choice or much control over what’s served, even though they know it doesn’t [meet the criteria for a healthy canteen]. (Name) says this is why the notes in PHIMS is important to help people understand the context of the area... She can’t work with the businesses, instead, she just talks to the principal and tries to provide some resources. She says, “You can’t work with them, you can’t do anything about that, and most of the time [the canteen practice] the only thing they’re not achieving due to circumstance.” – Team G
***Contextual variations***	
Site-level: Local community context	(Name) explained that the strategies that work for Sydney Metro are going to be hard to promote here. For instance, there are no footpaths in the rural areas and the children have to travel a long way to school. -Team F
Site-level: More pressing needs or different priorities	(This team) talks about how they try to align with schools health framework, and emphasize this. But schools have other issues to deal with. These issues include truancy. And “social welfare issues” that are rooted in history between place and Aboriginal communities. She tells a story of how she went to speak with a school about [the HCI program] and the principal told her that that day, he was dealing with 8 “displaced students who didn’t have housing.” – Team I
Site-level: Alignment between program aims and sites’ needs and/or priorities	[The Early Childhood Services program] is a totally different program from [the primary schools program], because the setting is so different. [The early childhood program] sits more easily in the child care services, it aligns more with what the child care centres already do, and [the practitioners] have something relevant to offer because the practices correspond to the requirements in their service agreements. The school environment is different and there is so much else going on. – Team J “I think ultimately, it’s about supporting each service in what they want to do. I think we’ve come into a little bit too much with a government perspective and pushing what we want them to do. Some of these, especially child cares, they’re private businesses, they don’t have to do what we say. So if we’re not supporting them to achieve their goals, we’re not going to get anywhere with them. It’s the same with the schools. The education used to be a 50–50 partnership between health and education and education just isn’t engaged. So, we’re trying to push something on them that doesn’t really fit into what they do. We’re not recognizing their capacity and their skills by doing that.” Interview in Team G
HCI-team level: Proximity of sites to Team offices	(This team) doesn’t often travel, the limit that they put on travel time is about 3.5 h (one way). 2 h is not considered a far drive for them. However, sometimes they will go out to schools but they must be strategic and maximize their travel as much as possible. – Team I
HCI-team level: Ratio of sites to practitioner	She questions how other teams can do it. Explains that their newest staff member came from (Team name) where she was responsible for > 300 sites. Here, they each have 50 sites per 1 FTE. With 300 sites, there is no way that they can do more than just focus on the practices. So to the new staff, coming here was a “health promotion dream.” – Team A
HCI-team level: Access to additional financial resources beyond HCI funding	[They have] no time to develop resources. “We get jealous when we see what other [teams] produce.” But they just don’t have time. Or money. Team G
***Incremental progress made over time***	
Accumulation of activities over a long period of time	(Name) was happy that he got the principal's permission to go directly to the educator, because the principal is too busy to make things happen, she just needs to agree and then he needs someone else to do the job. At this centre they have had so many changes a couple of years ago that they were not ready, so he just circled around and built a relationship, now they are getting there. 'That is just patience, just being there for when they are ready’. This is what the implementation plans from the ministry do not always get, it is about what our centre’s needs are, not what the program’s needs are. – Team L
Extensive time and effort to build relationships provide the foundation for HCI	I ask (name) how important the personal stuff is for her day-to-day work? (Name) thinks it is probably the most important because that relationship and having that communication between them and their job and what we’re trying to get them to do, they’re not going to listen to you at all if there’s no … if you haven’t built that relationship there’s no way they’re going to make any changes so being able to build that relationship and have conversations with them is probably the most important thing in terms of getting them to actually make changes … - Team E
***When to ‘tick’ a practice achievement in PHIMS***	
Some practitioners were conservative in assigning a ‘tick’ in PHIMS	(Name) walks through each performance indicator and is surprisingly (the ethnographer’s estimation) quite conservative with her “ticking” of the boxes. From (the ethnographer’s) impression of the (Director of the childcare service), she was quite insistent that they do physical activity and that they are “reporting” on a weekly basis to the families. I may have given them more ticks. But (name) explains that the physical activity is not “structured,” the food interactions aren’t “intentional,” and although they are reporting, they don’t have a quality improvement mechanism in place which (name) sees as the purpose of the Practice #15: Site monitors and reports achievements of healthy eating and physical activity objectives annually. Therefore, she does not give them full scores in these areas, and indicates the site could improve upon these areas. – Team H
Some practitioners took a more “liberal” approach	“I think that conversations around healthy food or sometimes a bit everyday food that happens over lunch time, I value that, and I would say that’s happening every day. Where some officers think that it needs to be very structured and it needs to be an activity. Where I think – and this is sort of where my issue with PHIMS is because if you were compliant with all the minimum adoption standards, you'd have a horrible report. You would have services not meeting, and it would be a really poor indicator of what services are actually doing, just because of the gaps in how you collect the data.” – Team F
When to tick a box and comparing interpretations amongst teams was common	(Name) says that their program adoption has gone up for the first time in a long time, and it is now (above 70%). She says that other teams are higher, but “we are harsher” on the selection criteria. She tells the team, “we can decide as a team if we want to relax on the criteria cause we have it on the hardest setting.” – Team F A problem with PHIMS is that they have to rely on teacher report for the practices. She thinks that, compared to other teams, they are quite “accurate” in their interpretation of what is going on in the schools. What this means is that they don’t necessarily rely on the teacher report, and they are strict in their interpretation of practice achievement. To her, this is reflected in the fact that their practice achievement on the canteen strategy is at 22%, while the state’s average is 50%. - Team M
***Practitioners reflect on PHIMS ability to capture “intensity”***	
Partial progress towards practice achievements is not accounted for in PHIMS	They say the problem with PHIMS is that all practices are considered to be equal to each other in the PHIMS system. But the “canteen KPI is massive”; a massive amount of work to achieve, while other practices aren’t as hard to obtain. Because of the implementation targets, they are unable to focus their work on the practices that are going to make the “most difference” in terms of health outcomes. This changes the way they make decisions regarding how they spend their money – Team I
Practitioners were concerned about the difficulties of capturing incremental progress over a long period of time	(Name) says, “there’s a massive disconnect between what comes up on PHIMS and what you’ve done to get that data. And it’s really hard to translate what you do when you go out and you have a conversation with someone face-to-face, and you talk to them about what they do day to day, and come back to the office and you just type into a computer.” – Team E (The practitioner) says you have to be a “pragmatist” and that it’s a long-term/organic process to get things done … (it’s an) incremental approach- get a new principal, you get her onboard, now she’s onboard, you get the Parents and Citizens to chip in money, a new canteen person comes on board. Researcher: And then years later you tick a box in PHIMS and it gets counted? Practitioner: Exactly, do you see what I’m saying? The tick in PHIMS is like the tip of an iceberg. It’s that tiny bit above the surface. And behind it is years of chatting, visits, gently urging, suggesting they go in this direction rather than that direction. - Team G
PHIMS does not document the particular activities taken to achieve an outcome	(Name) told me about the fact that what is captured in PHIMS is essentially a tick in a box, but leading up to that there has been fact sheets, conversations, a whole lot of other stuff, so PHIMS is “not a true reflection” of the work they do. – Team E (Name) told me about one particular tick to demonstrate how PHIMS falls short; to look into the kids lunchboxes is one simple activity and one tick, but to bring himself into the position where he has the trust and the position to get to look at the kids’ lunchboxes took him 15 visits - Team L “But [our activities] need to be recorded because … we have to justify our roles. When they were talking about the funding, they were trying to work out how many hours we spent supporting each school and I didn’t have any data to be able to say that. I can say that we do a site visit every year, but I have no idea how many emails we do at this school. So unless it’s all in there, we need to be able to say to support 100 schools, it takes us 1200 phone calls, 1300 emails and just be able to actually quantify that. There’s no way of doing that at the moment”. – Interview in Team G

Unless otherwise noted, excerpts are from qualitative fieldnotes, written in the first person by the researchers. Quotation marks are used to denote verbatim quotes from participants. Site and individual names have been de-identified

*PHIMS* Population Health Information Management System, *HCI* Healthy Children Initiative, *KPI* Key Performance Indicators

#### The difficulty of particular practices

The intensity of work involved in achieving a practice varied, with some practices requiring greater time and effort (See Additional file 2). Practitioners indicated that the hardest practices were those that were multi-component, where missing one component would result in the failure of that target. Several practices would involve environmental-level changes that were beyond the control of an individual practitioner to directly influence (e.g. the school provides a supportive environment for healthy eating).

#### Contextual variations

Contextual variations that influenced intensity represented three levels: the HCI-team, site, and individual practitioner level (See Additional file 4). Contextual factors interacted with each other resulting in variations in degrees of “intensity” of an individual activity or practice. We describe site and HCI-level factors in more detail.

##### Site-level

Practitioners discussed that local community contexts influenced how difficult an individual practice was to achieve. For example, access to fresh food or to a local food provider influenced the achievability of the healthy canteen practice. Some practitioners described that sites often had more pressing needs or priorities (e.g. truancy or homelessness) that superseded action on some HCI practices. The extent to which site needs and priorities aligned with the specific HCI program aims differed, with the general feeling that HCI aims aligned better with priorities of early childhood services than primary schools. Site-level staff turnover was also reported to affect how quickly progress could be made.

##### HCI team-level

**Fig. 1:**
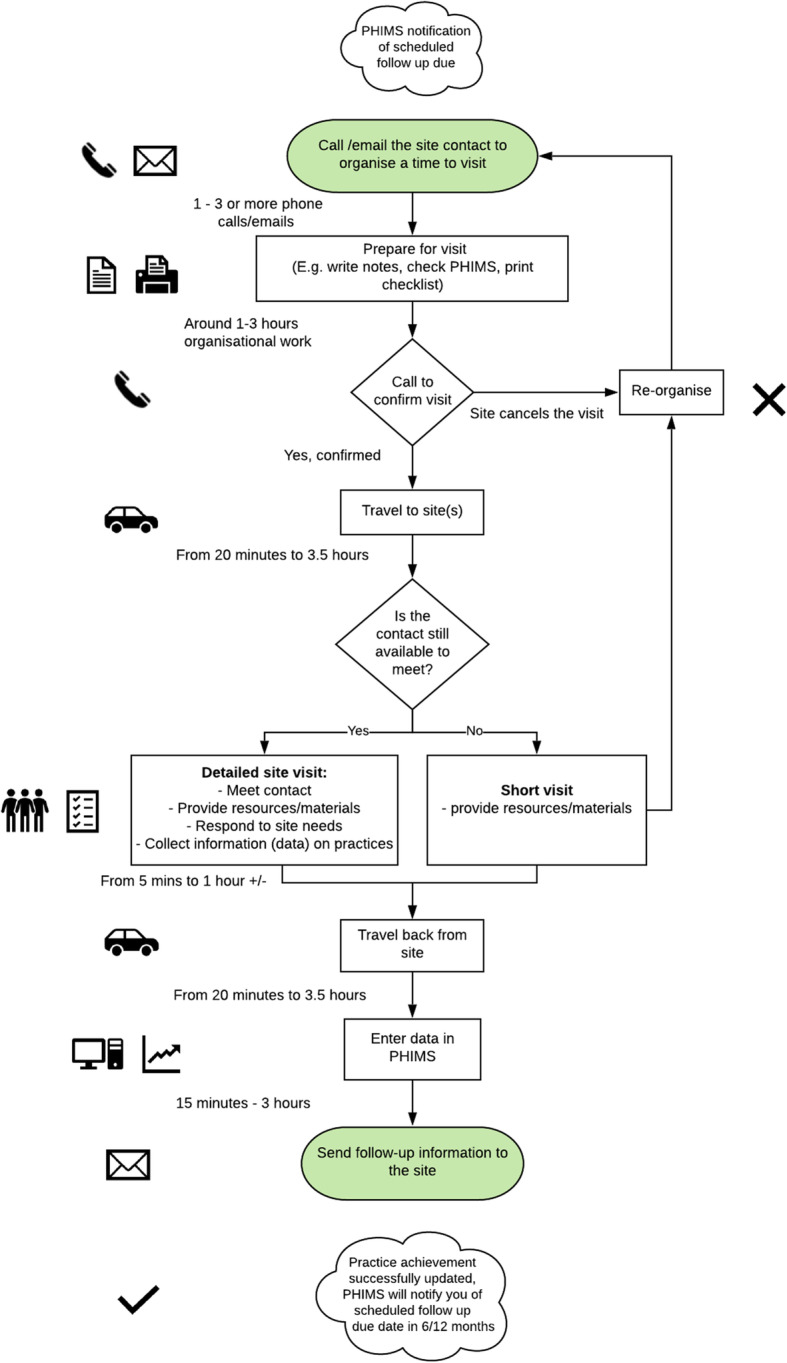
Schematic depicting variations in the intensity of work that goes into a site visit. Developed with LucidChart (free trial version) [26]

Participants described how geography, e.g. the rurality and size of districts and how dispersed sites were within it, influenced the time and effort involved in achieving a practice. The proximity of sites to offices varied by teams and ranged from a few minutes to travel between urban sites, to over 3.5 h to reach sites in remote areas. There was variation in the ratio of sites per practitioner between teams ranging from under 50 to over 150 sites per practitioner. High ratios negatively influenced the degree to which practitioners could spend time with sites. Practitioners described that HCI teams’ access to financial and staff resources influenced their ability to engage in innovative work. Much like the site-level factors, staff turnover in HCI teams was reported as affecting program delivery. In Fig. 1, we compiled observations across multiple practitioners completing the same task - a site visit - to illustrate how intensity of a task varies depending on the interplay between the site and aspects of the context in which HCI implementation occurs.

#### Incremental progress made over time

The intensity of work was described as an accumulation of activities over an extended period, sometimes years reflecting an extensive and prolonged commitment to building relationships between the practitioner and key site contacts. Many practitioners explained that relationships provided the foundation for HCI’s success, and therefore highly valued this work. Relationships were drawn upon to initially implement practices at a site, and to monitor progress over time. Some teams reported they had insufficient time or capacity to devote to relationship building, and that it limited their progress with sites.

#### When to “tick” an implementation target

**Fig. 2:**
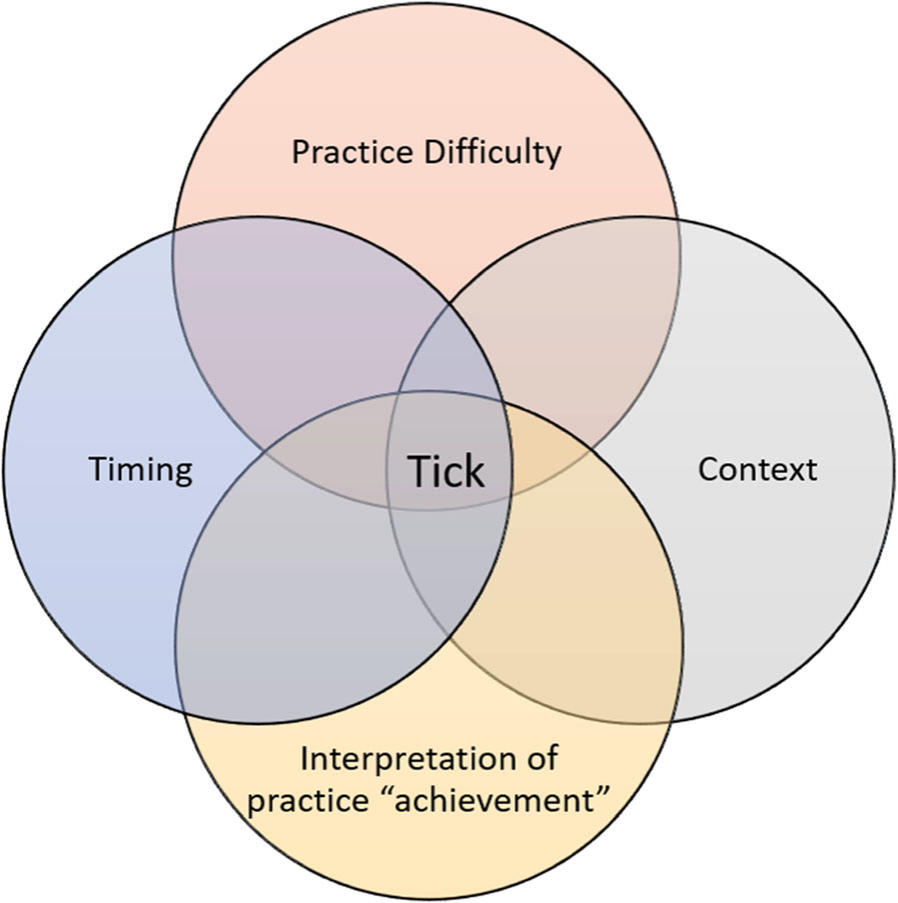
Factors overlap and influence the intensity of work involved in achieving a practice, i.e. a “tick” in PHIMS

Determining whether a practice was achieved or not, and therefore, whether it could be marked or “ticked” as achieved in PHIMS was source of much discussion amongst practitioners. For example, one practitioner described her approach as “conservative,” contrasting this with other practitioners she perceived as more “liberal” in their determination of whether a site had achieved a practice. We observed other practitioners making similar comparisons. Given the variations in context, degree of practice difficulty, the incremental nature of progress, and the range of individual interpretations, the achievement of a ‘tick’ in PHIMS was partly attributable to skilful implementation and partly serendipitous alignment with context (see Fig. 2).

#### How intensity is captured in PHIMS

Although practice reporting in PHIMS allowed practitioners to indicate how many “parts” of a multi-component target have been achieved, partial progress towards achieving a practice is not accounted for in the overall tallying of performance indicators. Practitioners were aware and concerned by the implications and difficulties of capturing such incremental – but hugely meaningful – progress over a long period of time in PHIMS. PHIMS was seen to be outcome-centric in that it does not document the particular activities taken to achieve an outcome.

## Discussion

To our knowledge, this is one of the first studies to examine the sustained use of an electronic performance monitoring system for health promotion implementation at scale. Given that very few electronic implementation monitoring systems and their uses are described in the literature, the insights gained from studying PHIMS provides novel considerations for the design of future systems. Such considerations are not limited to the design of the IT system itself, but also include the process of monitoring as well as the larger management context in which IT systems are used to govern large scale program delivery. These considerations are summarised in Table 4.

**Table 4:** Considerations for the design of future performance monitoring systems for health promotion implementation

**Considerations for the design of the IT system**	
• Mechanisms are needed to sort and retrieve information stored as “free text” data. Existing examples range from inbuilt search functions, quality audit processes, and machine learning/text mining methods.	
• Capturing data about behaviours of system users can be used to improve efficiency of design and inform decisions about how to use the system in practice. Examples could include information about time spent on individual functions, number of log-ins and time of day used, device used to log on (e.g. mobile vs computer), etc	
• Fields or data points could be inbuilt in IT systems to capture work that is outside of standardised program implementation. Such information- e.g. contact details, activity logs, distribution of materials, etc. – likely mirrors information required for delivery of the standardised program and could be made easily distinguishable, thereby harnessing and expanding upon existing infrastructure to serve multiple purposes	
• Provisions are needed to track and summarise the types of activities involved (i.e. breadth) and number of steps involved, and over what period of time (i.e. intensity), in achieving a target. In the context of PHIMS, this data is already being captured but requires mechanisms by which to better summarise, consolidate, and make existing data more informative and meaningful	
• Practitioners may be best-placed to identify key contextual indicators that influence target achievement and should be involved in identifying what data requires routine collection	
**Considerations for the measurement and monitoring of implementation and performance targets**	
• Track incremental progress toward target achievements outside notes function	
• In assessing progress, consider applying weights based on contextual indicators to account for variabilities in context that influence progress towards target achievement	
**Considerations for the broader management context in which IT monitoring systems are used**	
• Ensure further mechanisms and tools exist alongside the formal system to support innovative practice, and to capture and elevate innovations that develop alongside standardised programs	
• Incorporate audit and feedback tools and processes that provide useful strategies to use data for and facilitate quality improvement	
• Recognise and attend to tensions between performance monitoring functions and quality improvement functions. Adapt performance management structures to facilitate collaboration and innovation over competition	

Our analysis highlights ways in which a tick in PHIMS - indicating that practice change has been achieved - does not always convey the range and types of activities (i.e. breadth) involved in implementation, nor the time and effort (i.e. intensity) activities take. Program target achievement as reported by PHIMS reflects only the “tip of the iceberg” in terms of the scope of work employed, and importantly, some aspects of this work are better captured or represented than others.

### Implications for capturing breadth of implementation work

By studying PHIMS in practice, we uncovered a broader story behind the program monitoring data about the work that goes into implementing the flagship HCI programs. The 15 categories of activities we identified that constitute the breadth of HCI work reflect, in part, activities that have been identified elsewhere as program components [27]. But we also observed that teams engaged in varied activities that practitioners described as extending beyond HCI primary aims. Practitioners developed new materials and activities to complement HCI, but they also responded to local site needs, strengths and opportunities, or developed projects of particular interest to individual practitioners and their expertise. Sometimes this involved working to address non-obesity related health issues. That teams undertake “extra-HCI” tasks aligns with HCI’s overall management philosophy of “tight-loose-tight” – that is, local teams have discretion over how they achieve set implementation targets [28]. PHIMS, however, had no specific function to capture information about these activities. This lack of recording ability likely reinforced the feeling among practitioners that these tasks were “extra” or not part of HCI.

PHIMS is not the only mechanism by which HCI implementation is governed or managed at the state level. PHIMS sits within a broader management context in which it is one of many mechanisms used by NSW Health to monitor progress, support implementation, and recognise innovative practice. Practitioners are encouraged to share information and innovations at annual state-wide conferences, regular teleconferences and on an online portal. At the local level, informal technologies that sit alongside PHIMS (e.g. spreadsheets, paper files etc) are used by practitioners to capture and record non-HCI specific work, as well as new knowledge created about how best to implement the HCI program. We explore these methods elsewhere [22]. While these other mechanisms may provide better flexibility to capturing emerging and adaptive work, the inability of PHIMS to capture even simple information about extra work performed presents a potential missed opportunity.

It is not feasible or useful for IT systems to capture every aspect of practice, and we are not suggesting that they should. However, not capturing ‘extra’ work in the long term may pose problems because our understanding of a program, and what it takes to implement it well, may come to be defined only by what is monitored and measured via a recording system. Data from recording systems may therefore present a distorted view of implementation – one that defines and therefore standardises a program as the delivery of a particular set of tasks. When in fact, achieving successful implementation may require that practitioners undertake a range of activities that do not directly align with a program’s aims, but enable practitioners to build relationships and trust with local partners through which subsequent program implementation becomes possible. Consistently documenting information about extra work and new innovations in addition to documenting aspects of implementation and unexpected effects could provide information to demonstrate multiple benefits and effects of investing in health promotion work. The challenge, therefore, is to design IT systems that enable program implementation to remain ‘loose,’ while still capturing meaningful information about progress towards target achievement. Importantly, IT must be engineered in a way that allows for ongoing enhancements to enable the system to evolve as the context changes. Many of the limitations of PHIMS likely reflect changes in practice and the HCI program over time [29].

We also identified that some of the most valuable information about work with sites may be contained within PHIMS, but is currently inaccessible, buried within free-text note fields that are difficult to query or collate. This problem is not unique to population health systems, and much has been written about strategies used for “mining” such data from electronic health records [30]. In clinical contexts, audit and feedback processes as part of continuous quality improvement initiatives have shown promise as a means of effectively using data from electronic health records to improve health promotion services [31]. Audit tools assist practitioners to review records, systematically collect information – particularly from qualitative data – and use information to inform improvement strategies. As part of continuous quality improvement initiatives, teams undertake iterative cycles of reviewing and extracting data from records using audit tools, reviewing data to identify areas in need of improvement, then developing, trialling and testing a strategy to improve practice and improve implementation. Such improvement processes would be a useful complement to health promotion IT systems by providing a structured process through which teams can better harness and learn from qualitative notes and apply these learnings to practice improvement. Interestingly, one person in our study did develop a quality audit tool to review PHIMS records, but it was never finalised, implemented or otherwise shared beyond that team.

### Implications for capturing intensity of implementation work

We identified a confluence of four factors – practice difficulty, context, time, and interpretation – that must align to achieve a “tick” in PHIMS. However, almost none of these factors appeared to be captured by PHIMS. This was cause for concern among the teams, who debated when to “tick” a box - despite a standard monitoring guide to support decision making at this level being the part of program fidelity – and questioned whether these factors are accounted for when state-level decisions about HCI implementation are made.

Currently, PHIMS reports whether practices are implemented or not, but fails to capture or appreciate why implementation fails or succeeds. Information that could account for variation is not captured or collated in a meaningful way but could explain why target achievement looks different in different sites. If a lot of data are being recorded off the formal record, or extensive differences in workloads are being simply absorbed by the teams, then this deprives policy-level decision makers and IT designers of the opportunity to re-tweak the design of the scale up protocols, the funding and the recording of practice. Implementation scientists are concerned with developing measures by which to measure aspects of context, process and outcomes in implementation [32]. But given diverse contextual factors that influence implementation [33] comprehensive measuring of all these factors would be infeasible and onerous. Carefully selecting meaningful indicators is critical. Front-line practitioners are likely best-placed to recognise which indicators matter and why, given their deep understanding of local contexts.

Further, if monitoring systems are only concerned with whether a practice is achieved or not and fail to capture the incremental work processes accumulating in practice improvement, the system risks creating an artificial threshold effect wherein practitioners place higher value on completing work tasks that are 'counted,’ or, more easily contribute to practice achievement - rather than prioritizing those resulting in the greatest health improvements. Indeed, this issue is explored more fully in another paper from this research [21].

Indicators of incremental progress towards target achievement (i.e. process evaluation measures) are a simple addition that could improve PHIMS reporting formulas and future implementation monitoring systems. Similarly, summaries of the time and effort involved in achieving targets can likely be calculated using fields already existing in PHIMS (e.g. by tallying the number of notes entered, phone calls, emails and visits made to sites, graphs of progress over time, etc). The practices are at present “unweighted” – there are no extra points in achieving the hardest practices, but perhaps there should be. Such information would be useful in better understanding and recognising progress made over time. Given that other Australian jurisdictions have cut childhood obesity prevention programs, whereas HCI has been sustained [15], tracking progress over time would provide important documentation regarding how long-term investments are required to bring about the cultural and whole-of-environment change necessary to impact complex problems like obesity.

### Limitations

Given the short period of observations at each site, we were unable to observe practitioners performing all HCI activities. As such, our results are not meant to be an exhaustive list but rather a snapshot of the range of tasks conducted across the HCI teams at one point in time. Using a directed content analysis approach enabled us to identify and explore in our data the activities most relevant to our research questions, but may have missed relevant data that was not captured during the first coding pass. We minimized this risk by reading many fieldnotes in their entirety, by using researcher triangulation, and by ongoing connections and consultation with the field and with our partners to ensure that we had not missed any key activities. A thorough exploration of the *intensity* of implementation was precluded by our research design and reflects another limitation of this study. Ideally, such an exploration would adopt a longitudinal approach to explore how implementation builds over time whereas our observations were limited to only a few days with each team. From talking to practitioners, particularly those who are more experienced, we gained a sense of the variations in intensity across time and geographic location which we reported here. The researchers were conscious of producing ethnographic and sometimes challenging insights within the context of a policy and practice partnership, with partners having ongoing accountability for the system being studied. Other papers present our reflectivity about working in co-production and research impact [29, 34] (Loblay V, Conte K, Groen S, Green A, Innes-Hughes C, Milat A, et al. The Weight of Words: collaborative practice and the challenge of coproducing ethnography within a health policy research partnership, submitted).

## Conclusion

We set out to study implementation of prevention programs in process, and in doing so uncovered a rich story behind the program monitoring data. Our findings illuminate what is hidden beneath the surface, what work isn’t captured or reflected in PHIMS data, as well as, what is captured and potentially brought to focus. By understanding the full breadth of work and time investment required to successfully achieve a change in practice, we become better poised to capture and appreciate the value and multiple effects of investments in large-scale prevention initiatives. Insights gained through our ethnography illustrate several contextual factors that contribute to implementation outcomes that may be missed by implementation monitoring systems focused solely on outcome-centric indicators. Such insights highlight some of the possible benefits of harnessing the pragmatic knowledge of local practitioners, well positioned to assist policy makers to develop more accurate and meaningful performance indicators that are sensitive to diversity across contexts. Our results suggest that capturing the additional work that exists alongside standardised program implementation may yield new insights into the broad range of activities that exist around implementation efforts. Together, these findings provide implications to inform the design of future IT systems capable of tracking population-level prevention policies and programs.

## Supplementary information

**Additional file 1.** Additional information as per the consolidated criteria for reporting qualitative research (COREQ) checklist.

**Additional file 2 **Target practices for the Healthy Children Initiative programs, *Live Life Well@ School* and *Munch and Move®,* on which key performance indicator targets are based.

**Additional file 3.** Description of codes from project codebook extracted for this analysis.

**Additional file 4.** Summary of factors that influence the breadth and intensity of work in implementation.

## Data Availability

The datasets generated and/or analysed during the current study are not publicly available due to information that could compromise research participant consent and privacy.

## Abbreviations

PHIMS: Population Health Information Management System
IT: Information technology
NSW: New South Wales
HCI: Healthy Children Initiative
KPI: Key performance indicator
