# Supplementary material for: Can an electronic monitoring system capture implementation of health promotion programs? A focussed ethnographic exploration of the story behind program monitoring data
Source: BMC Public Health. 2020 Jun 12;20:917. doi: 10.1186/s12889-020-08644-2 (PMC7291504; doi:10.1186/s12889-020-08644-2)
Supplement: Supplementary file 1 — Additional file 1. Additional information as per the consolidated criteria for reporting qualitative research (COREQ) checklist. [file 12889_2020_8644_MOESM1_ESM.docx]

| **Appendix 1.** **Additional information as per the consolidated criteria for reporting qualitative research (COREQ) checklist** | |
| --- | --- |
| **Domain 1: Research Team and reflexivity** | |
| **Personal Characteristics**   1. Interviewer/facilitator; 2. Credentials; 3. Occupation; 4. Gender; Experience and Training | Ethnographic data collection was conducted by KC, VL and SG. KC is a public health researcher with 7 years experience and training in qualitative methods, and 11 years experience as a health promotion practitioner. She trained the other staff members and oversaw the fieldwork activities. VL and SG are trained ethnographers who collectively have 26 years experience. LM has a Master of Public Health and was employed as a research assistant by the University of Sydney to help support this work. All were employed by the University of Sydney, in roles funded by the Australian Prevention Partnership Centre - a collaboratively funded research centre that receives funds from the NHMRC and others. CIH has a Master of Public Health and a background in public health nutrition and is an experienced manager of population-level health programs. She and AG manage and support HCI implementation in their role with the NSW Office of Preventive Health, AG has over 15 years’ experience in health promotion practice with particular focus on childhood obesity. AM leads a team in NSW Health responsible for evidence and evaluation. He has long standing research experience in program scale-up and scalability. LP leads the population health information management portfolio at NSW Health, including the development of PHIMS. She has an engineering background and experience in ICT development and implementation. MW is a highly experienced health promotion director with over 20 years’ experience in leading one of the largest health promotion teams (including study participants) in NSW and has been involved in the development of PHIMS from its inception. ST is a senior executive at NSW Health with over 25 years experience in population and public health policy. At the time of the study, JM was a senior executive at NSW Health; she has 30 years experience in health promotion policy and implementation. Relevant to this project, the NSW Ministry of Health –whose employees are co-authors of this paper- is one funding partner of TAPPC. PH oversaw all study activities as guarantor. She is a former health promotion practitioner in NSW and public health researcher of 30+ years. |
| **Relationship with participants**  6. Relationships established; 7. Participant knowledge of the interviewer; 8. Interviewer characteristics | The process of synthesising the research design and study questions involved getting to know many of the research participants and consulting them as part of the design process. The study was designed and funded as a consequence of a research partnership formed between PH (university) and JM,ST, AM, AG, LP, MW and C I-H. (NSW Health). This design was further developed by KC in the early phase of fieldwork conducted by KC, one year prior to the in-depth ethnographic work on which this analysis is based. Ethnographic fieldwork also included many informal interactions like lunch, coffee, car trips that enabled more intimate relationships and personal discussions with researchers, beyond usual interview settings. Participants were informed that the researchers were working in partnership with decision-makers at the NSW Ministry of Health. To ensure confidentiality, they were reassured that only the researchers would have access to and view de-identified quotes, and that interpretations of data would be shared with them for feedback prior to presentation to the Ministry. |
| **Doman 2: study design** | |
| **Theoretical framework**  9. Methodological orientation and theory | The theoretical grounding of the overall study is outlined in detail in a protocol paper^1^. The methodological orientation was primarily ethnographic. |
| **Participant selection**  10. Sampling; 11. Method of approach; 12; Sample Size; 13. Non-Participation | At the site-level, all teams that implemented the Healthy Children Initiative agreed to participate. At the team level, one-hundred and six individuals agreed to participate of one-hundred and twenty-six identified individuals. Our sampling was opportunistic in that we only observed individuals that were present at the time of the observation, so some of the non-participants may not have been present and observed. No HCI team member declined participation, but several did not return their consent forms. KC met with most sites in-person or via phone to discuss the study purpose prior to fieldwork, and followed up with written documentation of study information and consent. At the time of fieldwork, researchers discussed the study objectives with participants and answered questions. Individuals had the opportunity to provide us with their decision to consent privately. |
| **Setting**  14. Setting of data collection; 15. Presence of non-participants; 16. Description of sample | Data collection occurred in multiple places including at the local health district’s offices, riding in cars to site visits, at site visits, at meetings and via phone. Fieldwork visits ranged from 1-5 days. We informed non-participants about the research and that we were documenting our observations, and assured them we would not collect any detailed information about them. |
| **Data collection**  17. Interview guide; 18. Repeat interviews; 19. Audio/visual recording; 20. Field notes; 21. Duration; 22. Data saturation; 23. Transcripts returned | KC developed a field note template and revised it in collaboration with SG and VL to create a structured debriefing process to ensure consistency among researchers. Extensive field notes were recorded as soon as possible following the fieldwork. On occasion, and when granted permission, we audio recorded brief interviews or team meetings. These recordings were transcribed verbatim by a third party and checked for accuracy. Given the nature of our approach, obtaining data saturation was not possible. Rather, by including all sites we ensured the widest range of possible experiences and viewpoint. Except for a few instances to double-check our interpretations, we did not share our field notes with participants. |
| **Domain 3. Analysis and findings** | |
| **Data analysis**  24. Number of data coders; 25. Description of the coding tree; Derivation of themes; 27. Software; 28. Participant checking | KC, VL and SG developed an initial coding structure, in consultation with PH. KC, VL and SG conducted coding for all field notes; each was coded by two researchers in NVIVO qualitative data management software^2^. From this initial codebook, a subsequent and more detailed coding analysis for this paper was conducted by LM, guided by KC. KC and LM discussed emerging themes on an ongoing basis with VL, SG, and PH, and ideas were consolidated and presented to the wider team for feedback. The coding and theming process is discussed in detail in our manuscript. |
| **Reporting**  29. Quotations presented; 30. Data and findings consistent; 31. Clarity of major themes; 32. Clarity of minor themes | We report our results using quotes extracted from the field notes that are illustrative of both major and minor themes. Consistency between data and findings were iteratively checked throughout the analysis, to ensure that consistency and accuracy between the two. |

*1. Conte KP, Groen S, Loblay V, et al. Dynamics behind the scale up of evidence-based obesity prevention: protocol for a multi-site case study of an electronic implementation monitoring system in health promotion practice. Imp Sci. 2017;12(1):146.*

*2. Nvivo qualitative data analysis software. 11 ed: QSR International Pty Ltd.; 2015.*
