# Supplementary material for: Can an electronic monitoring system capture implementation of health promotion programs? A focussed ethnographic exploration of the story behind program monitoring data
Source: BMC Public Health. 2020 Jun 12;20:917. doi: 10.1186/s12889-020-08644-2 (PMC7291504; doi:10.1186/s12889-020-08644-2)
Supplement: Supplementary file 2 — Additional file 2 Target practices for the Healthy Children Initiative programs, Live Life Well@ School and Munch and Move®, on which key performance indicator targets are based. [file 12889_2020_8644_MOESM2_ESM.docx]

Appendix 2: Target practices for the Healthy Children Initiative programs, *Live Life Well@ School* and *Munch and Move®,* on which key performance indicator targets are based

| ***Live Life Well @ School* practices** | ***Munch & Move* practices** |
| --- | --- |
| Practice 1: The school provides curriculum leaning experiences regarding healthy eating and physical activity and sedentary behaviour | Practice 1: Service monitors food and drinks that are in children’s lunchboxes every day |
| Practice 2: The school explicitly addresses fundamental movement skill development as part of the Personal Development, Health and Physical Education programs. | Practice 2: Service menu includes fruit and vegetables at least once per day |
| Practice 3: The school provides the opportunity for classes to eat fruit, vegetables and fruit and drink water | Practice 3: Service menu includes only healthy snack options every day |
| Practice 4: The school encourages physical activity during recess and/or lunch. | Practice 4: Service supplies age appropriate drinks every day |
| ***Practice 5*: The school provides a supportive environment for healthy eating. (canteens, school activities involving food and drink).** | Practice 5: Service provides structured and specific learning experiences about healthy  eating at least 2 times per week |
| Practice 6: The school provides information to families on healthy eating, healthy lunchboxes, physical activity and limiting small screen recreation. | Practice 6: Service provides tummy time for babies 0-12 months of age every day |
| Practice 7: Teaching staff are provided with professional learning/ development to promote healthy eating and physical activity to students | Practice 7: Service provides physical activity for 1-5 year olds at least 25% of the daily  opening hours |
| Practice 8: The school has an identified team/committee with executive membership to support the implementation of Live Life Well @ School or similar initiatives. | ***Practice 8*: Service provides fundamental movement skills for children 3-5 years of age every day, to at least 90% of children** |
| Practice 9: School planning processes (e.g. strategic, annual, operational plans) incorporate *Live Life Well @ School* strategies | Practice 9: Service use of small screen recreation by 3-5 year olds is appropriate |
| Practice 10: The school monitors and reports annually on the implementation and outcomes of *Live Life Well @ School* strategies. | Practice 10: Service has a written nutrition policy |
|  | Practice 11: Service has a written physical activity policy |
|  | Practice 12: Service has a written policy restricting small screen recreation |
|  | Practice 13: Service provided health information to families within past 12 months |
|  | ***Practice 14*: Service has at least 50% of primary contact educators trained in nutrition and at least 50% trained in physical activity** |
|  | Practice 15: Service monitors and reports achievements of healthy eating and physical  activity objectives annually |
| ** Denotes practices identified by practitioners as the more challenging practices to achieve.* | |
