# Supplementary material for: Can an electronic monitoring system capture implementation of health promotion programs? A focussed ethnographic exploration of the story behind program monitoring data
Source: BMC Public Health. 2020 Jun 12;20:917. doi: 10.1186/s12889-020-08644-2 (PMC7291504; doi:10.1186/s12889-020-08644-2)
Supplement: Supplementary file 3 — Additional file 3. Description of codes from project codebook extracted for this analysis. [file 12889_2020_8644_MOESM3_ESM.docx]

Appendix 3: Description of codes from project codebook extracted for this analysis

| **Code** | **Description** |
| --- | --- |
| Tick | The work that it takes to achieve a "tick" |
| Key Performance Indicators |  |
| Assessment and interpretation of KPIs/practices | How do participants determine what "success" of KPI/practices look like? What do they think the KPIs are meant to achieve? How well do the KPIs lend themselves to interpretation? |
| The hardest KPI/practice | What is the hardest practice to achieve for each team? |
| Status of KPI achievement | Performance on KPIs, what's reflected in the graph, feelings and perceptions about their status |
| Live Life Well @ School vs. Munch & Move | Comparisons between the two flagship HCI programs that PHIMS monitors |
| HCI Team Activities |  |
| Work with sites not aimed at KPI/practice achievement | Going beyond KPI/practice achievement with a site, or work that's not aimed at achieving a particular KPI |
| Strategies | How the team makes decisions, weighs options, plans and uses resources to achieve implementation targets. Could include strategies that they say or think don't work. |
| Team work and communication | How HPOs work together, or not. Including communication |
| Relationship building with sites | What are techniques, strategies, approaches that HPOs use to initiate, foster, sustain relationships with people at sites |
| Knowledge creation and innovation | Examples of knowledge developed at the local level, may include research or evaluation, also innovations on the program or on other elements of practice |
| HCI Materials and resources | Materials/resources used and/or created to help the team in their delivery of HCI program; materials given to sites by practitioners |
| Data Entry | Observations of people entering data into PHIMS |
| Scheduled Follow-Ups | Scheduled follow-ups are a function and PHIMS, but the importance and relevance of them to practice and to accountability is contested. They pose a problem for staff. |
| Note: PHIMS=Population Health Information Management System; HCI=Healthy Children Initiative; KPI=Key Performance Indicator | |
