# Supplementary material for: Can an electronic monitoring system capture implementation of health promotion programs? A focussed ethnographic exploration of the story behind program monitoring data
Source: BMC Public Health. 2020 Jun 12;20:917. doi: 10.1186/s12889-020-08644-2 (PMC7291504; doi:10.1186/s12889-020-08644-2)
Supplement: Supplementary file 4 — Additional file 4. Summary of factors that influence the breadth and intensity of work in implementation. [file 12889_2020_8644_MOESM4_ESM.docx]

Factors that influence the breadth and intensity of work in implementation
